# An Ishmalite

**Published:** 1888-06-16

**Authors:** Leslie Keith

**Affiliations:** Author of "The Chilcotes," "Uncle Bob's Niece," "East and West," etc.


### AN ISHMAELITE.

By LESLIE KEITH,

Author of "The Chilcotes," "Uncle Bob's Niece," "East and West," etc.

(Continued from p. 169.)

CHAPTER X.—GETTING USED TO IT.

How did the new assistant prosper? Arthur's thoughts were with him every hour of that first week when he forced himself to stay away from Bermondsey in order that Fred might shake down in the new quarters. Arthur himself was dull enough over the defeat of his schemes, though he still had a hope that with time on his side he would prevail.

Mr. Eastgate eyed him furtively on his return. He asked no questions, and he invited no confidence on Arthur's part, but he was very moody and ill-tempered, and so generally difficult to please that he must have tried the patience of any housekeeper less placid than Miss Ella Coates. She on her part received Arthur back again with the most unruffled good humour.

"I knew you would come back, Arthur. I knew you would think better of this foolish idea of going abroad," she said. "I am glad I did not write to mamma——"

"Your mamma would certainly not have hindered me," said Arthur, with some heat and haughtiness. "And because I have changed my plans in the meantime, you need not suppose that I have given them up."

"You should not be so violent, Arthur," said Ella mildly; "it is very bad taste." Ella herself was never violent, and indeed her perfect composure and ladylike calm irritated him and checked his wish to tell her all that was in his heart. Could you confide in an iceberg and expect it to reciprocate?

Arthur's love had come to a dangerous pass when he flung out of the drawing-room where Ella sat composedly drawing her needle in and out, or playing the tinkling music which could not lure him back to her side. He took his seat in the office once more, his uncle suffering him grimly, but all Arthur's thoughts were centred in the little shop across the river. When he came home to the big house and sat through the long, solemn dinner, his mind and imagination were in the stuffy, low-roofed room in the dingy, busy street. He wished himself there; his heart was with Fred the sinner, whom he loved better than the girl he had pledged himself to marry. Ella had no sympathy with sinners, though she called herself a miserable one every Sunday. She believed in virtue, and talked of it as if it were a kind of portable property, chiefly the possession of the rich.

The new assistant's thoughts very likely went back often enough to the old home he had vowed never to see more. But he did not allow them to influence his work. He had brought back the habit of a dogged mechanical application with him from Dartmoor, and he filled all his hours with a diligence that left him scarcely more liberty than he had enjoyed when behind those guarding walls. He was free to go where he would, but he never crossed the threshold of the little shop. He fell into the ways of the strange household without seeming difficulty, but he could not help knowing that his sombre, silent looks were a restraint upon its cheerfulness. When he was summoned to the family dinner in that long low room, Delia's saucy nonsense was hushed, and the familiar jests were not produced. Miss Betsy regarded the newcomer with dark disfavour, and there was another member of the family who took no more pains to conceal his dislike.

This was a sickly and deformed young man, who acted as clerk and book-keeper in the eating-house, and took the customers' pence. Tucker did not hold his tongue, and used it very freely when the new librarian was out of hearing.

"What does the feller want here?" he demanded. "He's a swell, that's what he is; he can't deceive G. Tucker, and when a swell comes down 'ere, and pretends to 'umble himself to work like this, you take my word for it there's something fishy about the business."

"He's a gentleman, as anyone can see, if he's a poor 'un. I don't think his 'ealth is good——"

"Health," corrected the stern Miss Betsy.

"Well, well, Betsy," said the easy Mrs. Proudie, "you can't be a-changing of me at my age; I ain't 'ad schoolin' like you 'ad——"

"You don't need to tell me that, Barbara Proudie," said her sister-in-law loftily; "as for you, Tucker, it would be more to your credit if you copied the new young man in his diligence, than used your ill tongue on him when his back was turned. Your master has been gone this ten minutes or more, and you're still havering here."

Thus, while Miss Betsy sternly held Fred at arm's length in those few moments he spent upstairs, she would not permit him to be maligned in his absence by the spiteful little hunchback, who hated any man possessed of a straight spine. Perhaps he hated Fred the more because of the opportunities his occupation gave him of being much in the company of Delia.

The possession of a puny body does not prevent a man from harbouring very strong passions—jealousy and love, to wit. The poor little deformed man was torn with jealous pangs as he sat at his high desk amid the steam of the cooking, and took the workmen's pence; with rage as he thought of pretty Delia behind the dividing wall, helping the "swell" to keep shop. He might have spared his fears, for Eastgate probably never so much as knew that the girl was pretty. He had scarcely looked at her, and she was little in the shop, being kept much upstairs to help her mother and aunt. It was the elder girl, Barbara, who was his assistant. Together they unpacked and arranged the books which Arthur had begged leave to choose. Arthur had sent down such a liberal collection of juvenile and grown-up literature that the little shop was turned into a miniature Mudie's. All the new shelves were used, and more had to be put up. Presently the back room was put into requisition too, and the gay bindings gleamed out with a friendly twinkle from dark corners.

Fred unpacked and worked unremittingly. When Delia came down to summon her sister to dinner he excused himself from going too on the plea that he wished to finish the classification of one bookcase before resting; and the girls went up alone.

"Where's Mr. Eastgate?" said the cripple, with a spiteful emphasis on the Mr.

"He's busy, and he doesn't want to come," said Delia shortly. "He has got all the books that ever were written, I believe, round him."

"Perhaps he would like a valet to wait on him, and a special table spread for his 'ighness."

"And it is no more than he would have a good right to," said Proudie, with stern reproof. "You stick to what you know about, Tucker, and keep a decent tongue in your head, if you please. Wife, you'll see that Mr. Eastgate gets a bit of dinner comfortable when he's ready for it."

"When his 'ighness is pleased to ring," muttered the cripple, but he did not dare to grumble aloud.

It was Barbara who spread the tray and carried it down to her coadjutor. Delia had offered her services, but the ever watchful Aunt Betsy interfered.

"You've got to help me wash up," she said. "Can't you see that your mother is tired?"

"Well, I ain't much good," said the placid Mrs. Proudie, who suffered from chronic weakness of the chest, "but I can dry if you give me a clean towel, Del. Here, Barbara, my dear, you'd better take some bottled hale to the pore gentleman downstairs. There must be dust enough in 'is throat with all them books."

"Wouldn't his 'ighness like champagne better?" cried the jealous little Tucker, hovering near Delia, and lingering vainly for a kind word or look from her.

"There's sherry wine in the press," said the grim Miss Betsy, withering the cripple with her glances. "Wait, girl, till I get you a wine glass."

Barbara took the tray down the winding staircase, and set it on a remote corner of the counter. Then she arranged a small table in a quiet nook, and spread the viands neatly on it. Fred was kneeling by a chest and taking out the books from it, and he had not looked up at her entrance.

She went over to him with a light step, and stood watching him for a moment in silence, then she said:

"Mr. Eastgate, won't you please stop for a few minutes? I am sure you must be very tired. I've brought you something to eat."

He looked up with a start. They had worked together all the morning and had scarcely exchanged a word. Barbara's voice was a very quiet and pleasant one, and something in its tone touched him, he scarce knew why. "Thank you, I am not tired, but I suppose one must eat."

"I'll take out the books if you'll tell me what to do."

He was about to say something, but he checked himself, and showed her what he was doing. She listened attentively, but she followed him to the little table in the corner.

"There's some wine," she said; "please take some. It was aunt Betsy who asked me to bring it."

"Miss Betsy Proudie?" He could not conceal his surprise. "I—I thought she hated me."

"Oh, no," smiled the girl, soberly; "it is her way. She is like father, she is very good."

"Your father is very good," said Fred gravely. The wine was certainly of an obscure vintage, and somewhat fiery, but he would have swallowed it after that if it had been poison.

When the books were all up on the shelves and duly catalogued, the assembled family came to see the altered shop. Mr. Proudie's honest heart was filled with delight and pride.

"There's not a shop to beat it, not within miles," he said. "There'll be a perfect 'Shirra-muir' on a Saturday night when the lads and lasses come with their pennies."

"Have you read them all?" asked Delia, looking with bright shy glances at the librarian.

"Oh no, not all."

"Well, here's two that will soon beat you," said Mr. Proudie, encircling a daughter with each arm, and looking at them with pride. "I've given my girls a good eddication, Mr. Eastgate; they've had a good bit of schooling, and for readers they're not to be beat on this side the water; and here's my wife will tell you the same."

"Well, they ain't showy," responded their mother; "if Del had had music——"

"The pianny? Nonsense; what does she want with the pianny-forte? She can read and write and sew her seam, and sing like a lintie, and what more does a young lass want? And here's books enough to keep them both going for the rest of their lives."

"Are we to die when we've read them all?" asked Delia, looking up and laughing. "If that's what you mean, father, I won't begin."

"There's more where these came from," said her father, pinching her ear. "You may live to be an old wife, lass, before you've done with the books."

More where these came from? Was it the young prince who sent them? And what a wonderful person he must be to know just what to choose! Arthur's task had been easy enough, and he could have filled the shop many times over, since fortunately the stream of good and healthy literature is quite as copious as the bad. It had grieved and outraged the heart of the honest Scotchman to observe as he did with what vile and immoral rubbish the young folks in the streets and courts around him filled their minds and imaginations.

This gutter literature is a thing apart, and is never seen or heard of in politer quarters. It is printed and published—who knows where? It is never met with at respectable bookshops or on the railway stalls, yet it has a vigorous circulation and the authors who write for it have a public to listen to them, for whose tastes they cater. Even when the tone is decent—and this is by no means always the case—what false and distorted views of life are set forth in those vulgar pages; what highly-spiced and sensational and impossible incidents are related there! The School Board has made it its concern that all boys and girls shall be taught to read, but when one sees with what inadequate rubbish they feed their eager young fancies, one is tempted to wish for the old comfortable, painless ignorance.

"Unless you can show them a better way," said Proudie, who had long cherished this ambition and was delighted to see it now within his grasp. He was the pioneer of the free library, and sought to do its work in this neglected corner. He saw the claimants flocking in and the books going out and passing from hand to hand. If they got despoiled of their first splendour and grew dingy, dog-eared, and greasy, what did it matter so long as they were read?

"And they'll bring custom to the shop, too." He would not have been a Scot if he had not cannily foreseen that comfortable contingency. It was mostly for philanthropy, but it was a little bit for John Proudie, too.

Miss Betsy, however, with the national dislike of waste strongly developed, came downstairs one day to the shop armed with a ream of brown paper and a large pair of shears.

"I am going to cover those books," she said severely to the librarian, who was busy with his catalogue. "Hand them down, if you please young man, one by one."

Fred obeyed the mandate silently. This grim and bony old woman had a right to command him. There was perhaps something morbid in his condition; he was crippled, too, like the little angry clerk next door, but his exception from ordinary conditions was of his own doing. His own doing—it was that that gave life to his bitterness and made him long for any kind of work that should weary him and stupefy his pain. *(To be continued.)*

